# Improving PARP inhibitor efficacy in high-grade serous ovarian carcinoma: A focus on the immune system

**DOI:** 10.3389/fgene.2022.886170

**Published:** 2022-09-09

**Authors:** Nirashaa T. Bound, Cassandra J. Vandenberg, Apriliana E. R. Kartikasari, Magdalena Plebanski, Clare L. Scott

**Affiliations:** ^1^ Cancer Biology and Stem Cells, Walter and Eliza Hall Institute of Medical Research, Parkville, VIC, Australia; ^2^ Cancer Ageing and Vaccines (CAVA), Translational Immunology & Nanotechnology Research Program, School of Health & Biomedical Sciences, RMIT University, Bundoora, VIC, Australia; ^3^ Department of Medical Biology, University of Melbourne, Parkville, VIC, Australia; ^4^ Peter MacCallum Cancer Centre, Parkville, VIC, Australia; ^5^ Royal Women’s Hospital, Parkville, VIC, Australia

**Keywords:** high-grade serous ovarian carcinoma, poly ADP-ribose polymerase inhibitors, epigenetics, immunotherapy, combination (combined) therapy, clinical trials, checkpoint inhibition, PARPi combinations

## Abstract

High-grade serous ovarian carcinoma (HGSOC) is a genomically unstable malignancy responsible for over 70% of all deaths due to ovarian cancer. With roughly 50% of all HGSOC harboring defects in the homologous recombination (HR) DNA repair pathway (e.g., *BRCA1/*2 mutations), the introduction of poly ADP-ribose polymerase inhibitors (PARPi) has dramatically improved outcomes for women with HR defective HGSOC. By blocking the repair of single-stranded DNA damage in cancer cells already lacking high-fidelity HR pathways, PARPi causes the accumulation of double-stranded DNA breaks, leading to cell death. Thus, this synthetic lethality results in PARPi selectively targeting cancer cells, resulting in impressive efficacy. Despite this, resistance to PARPi commonly develops through diverse mechanisms, such as the acquisition of secondary *BRCA1/2* mutations. Perhaps less well documented is that PARPi can impact both the tumour microenvironment and the immune response, through upregulation of the stimulator of interferon genes (STING) pathway, upregulation of immune checkpoints such as PD-L1, and by stimulating the production of pro-inflammatory cytokines. Whilst targeted immunotherapies have not yet found their place in the clinic for HGSOC, the evidence above, as well as ongoing studies exploring the synergistic effects of PARPi with immune agents, including immune checkpoint inhibitors, suggests potential for targeting the immune response in HGSOC. Additionally, combining PARPi with epigenetic-modulating drugs may improve PARPi efficacy, by inducing a BRCA-defective phenotype to sensitise resistant cancer cells to PARPi. Finally, invigorating an immune response during PARPi therapy may engage anti-cancer immune responses that potentiate efficacy and mitigate the development of PARPi resistance. Here, we will review the emerging PARPi literature with a focus on PARPi effects on the immune response in HGSOC, as well as the potential of epigenetic combination therapies. We highlight the potential of transforming HGSOC from a lethal to a chronic disease and increasing the likelihood of cure.

## 1 Introduction

Even with years of research and the development of a new effective therapy, high-grade serous ovarian carcinoma (HGSOC) remains, to this day, one of the most lethal gynaecological malignancies. HGSOC belongs to the type II class of epithelial ovarian cancers (EOC) and mostly develops from fallopian tube secretory cells into aggressive high-grade tumours with early metastatic potential (Pavlidis. et al., 2021). In contrast, type 1 EOC, such as endometrioid OC, is relatively indolent and genetically stable, with a better prognosis, arising from precursors such as endometriosis (McCluggage, 2011). Responsible for over 70% of all ovarian cancer (OC) deaths, only 30% of women affected with HGSOC are expected to survive five years (Dion et al., 2020). Current treatment for HGSOC includes a complete resection of the cancer and platinum/taxane chemotherapy, however, only 30% of women will remain in remission following this, with the remainder undergoing more chemo-resistant relapse occurring within 4–16 months (Agarwal and Kaye, 2003; Cooke and Brenton, 2011; Vaughan et al., 2011; Korkmaz et al., 2016). This high mortality rate is largely due to late-stage diagnosis and disease recurrence (Dion et al., 2020). The intra-tumoral heterogeneity that arises within HGSOC, enables the acquisition of resistance mechanisms to first-line treatments (Milanesio et al., 2020). Thus, there have been efforts to improve the first-line regimen, to introduce additional therapies, particularly in the maintenance setting, to combat recurrence, in order to improve outcomes for women with HGSOC.

Women who have received first-line therapy can be stratified into having platinum-resistant or platinum-sensitive HGSOC/OC (defined as women whose cancer progresses within six months or after six months respectively) (Lee and Matulonis, 2020). However, the fifth Ovarian Cancer Consensus Conference (OCCC) convened by the Gynecologic Cancer Intergroup (GCIG) in Tokyo, Japan in 2015 concurred that, “as time since last platinum chemotherapy represents a continuum of probability of response to further chemotherapy, a fixed 6-month cut-off decision on platinum sensitivity was neither sensible nor biologically relevant” suggesting a greater degree of flexibility should be taken into account when considering a patient’s treatment options (Colombo et al., 2019). Upon recurrence, platinum-sensitive OC continues to be treated with a platinum-based chemotherapy regimen, with combination platinum regimens having a better OS when compared with single-agent carboplatin, with a median overall survival (OS) of around 30 months (Lendermann et al., 2003; Lee and Matulonis, 2020; Vanacker et al., 2021). In the ICON7 trial (NCT00262847), the addition of the anti-angiogenic drug, bevacizumab (BV), to the platinum/taxane chemotherapy combination offered a slight extension of progression-free survival (PFS) for these women and of OS in those at high-risk for disease progression (Perren et al., 2011). However, regardless of treatment, most HGSOC patients relapse, with the degree of benefit derived from treatment and duration of remission decreasing with each subsequent line of treatment (Lee and Matulonis, 2020). Women with platinum-resistant OC are treated with non-platinum chemotherapies such as pegylated liposomal doxorubicin (PLD), weekly paclitaxel, gemcitabine, topotecan or oral cyclophosphamide. These non-platinum-containing regimens are comparable in terms of efficacy and typically have poor response rates, as low as 10%–15% with median OS of 12 months (Lheureux et al., 2019a; McMullen et al., 2020). In the AURELIA trial (NCT00976911), the addition of BV, to these second-line and beyond lines of chemotherapy increased the PFS of patients from 3.4 months to 6.7 months, however, there was no significant improvement in OS compared with chemotherapy alone (Pujade-Lauraine et al., 2014; Lee and Matulonis, 2020).

In 2014, the use of poly(ADP-ribose) polymerase inhibitors (PARPi) was approved for the treatment of recurrent, advanced *BRCA1/2-*mutant HGSOC (George et al., 2017). Within four years, phase III clinical trials of PARPi in the relapsed setting (SOLO2/ENGOT-Ov21 (NCT01874353), ARIEL3 (NCT01968213) and NOVA/ENGOT-OV16 (NCT01847274)) demonstrated improved PFS for women with either mutated or wild-type *BRCA1/2* (Tomao et al., 2019; Banerjee and Lord, 2020)*.* This led to the use of PARPi as a maintenance therapy regardless of *BRCA* status in recurrent OC, and subsequent phase III first-line trials (SOLO1 (NCT01844986), PAOLA-1/ENGOT-ov25 (NCT02477644), PRIMA/ENGOT-OV26/GOG-3012 trial (NCT026555016) and VELIA/GOG-3005 (NCT02470585) ) led to PARPi’s more recent use as a front-line maintenance therapeutic for women with mutant *BRCA1/2* (both germline and somatic) and then in the setting of platinum-responsive or HR defective (HRD) HGSOC (HRD status determined through tests such as the Myriad MyChoice™ test) (Banerjee and Lord, 2020)*.*

This was a major advance, as PARPi was the first targeted treatment approved for women with HGSOC which was dependent on certain genetic mutations being present in the cancer itself. However, the presence of specific HRD gene mutations have been concluded by recent phase III trials to not be essential, rather to predict which women will benefit from experiencing the strongest responses to PARPi therapy and summarised in a meta-analysis of a trial in relapsed OC (Coleman et al., 2017; Lee et al., 2019; Ray-Coquard et al., 2019; Matulonis et al., 2021). Treatment with PARPi offers a significant benefit to women, however acquired resistance has driven the requirement for the development of combinatorial therapeutic approaches, as women treated with single agent PARPi may develop recurrence which is resistant to both subsequent PARPi and to chemotherapy (Park et al., 2022). Extensive research has been performed to characterize the effects and mechanisms of action of PARPi. This has better defined which women with HGSOC would derive the most benefit from PARPi, including as single agent therapy, and continues to improve the likelihood that more women who are more likely to need PARPi combination therapy will be identifiable so that they can receive it. Additionally, this characterisation has demonstrated the effects of PARPi beyond its role as a DNA repair inhibitor, such as in inflammation and checkpoint expression, illuminating new pathways for combinatorial therapeutic approaches (Shen et al., 2019). This review summarizes the actionable mechanisms of PARPi in relation to HGSOC, highlighting effects on immune responses and epigenetic modulation, as well as relevant combinatorial clinical trials of PARPi.

## 2 Genomic and immune characteristics of HGSOC

**FIGURE 1:**
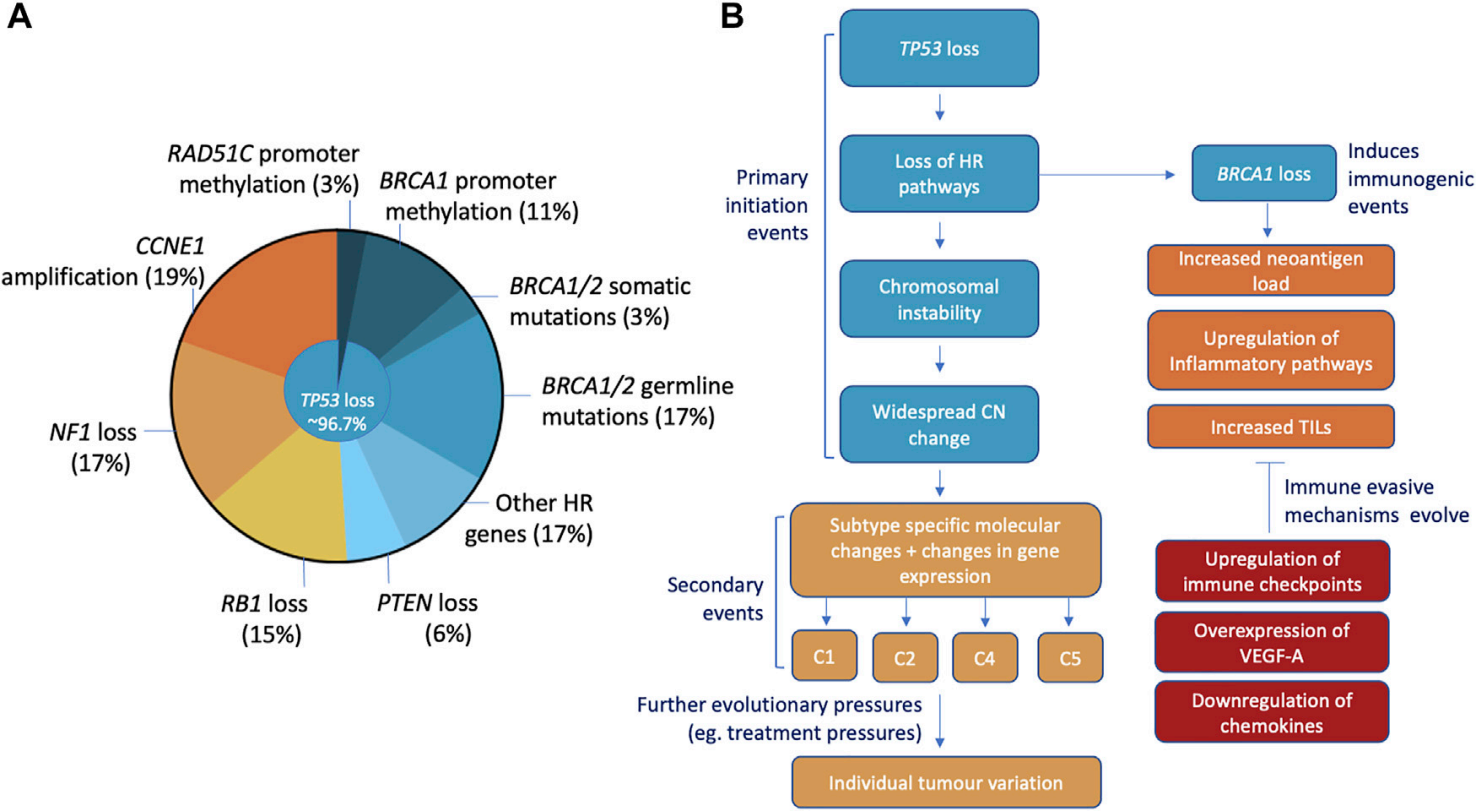
Characteristics, initiation, and molecular progression of HGSOC. **(A)** Most common mutations include ubiquitous loss of *TP53* (96.7% of cases), loss of *BRCA1/2* (somatic/germline mutations, promoter methylation), *CCNE1* amplification, *NF1*, *RB1* and *PTEN* mutations. **(B)** The loss of *TP53* is thought to be the initiating event that with subsequent loss of HR pathways stimulates the chromosomal instability and widespread copy number changes seen in HGSOC. This causes changes in gene expression and promotes the development of specific molecular changes that define the 4 HGSOC subtypes (C1, C2, C4, and C5). Loss of HR, specifically *BRCA1,* can elicit immune responses through increased neoantigen loads and upregulation of inflammatory pathways. Additionally, HRD and *BRCA* mutant tumours have been associated with elevated levels of TILs. Common immune evasion mechanisms that HGSOC develop to negate these innate immunogenic traits include the upregulation of immune checkpoints, overexpression of angiogenesis factor VEGF-A and the downregulation of immune-stimulating molecules.

In order to improve outcomes for PARPi, we must first understand the disease. HGSOC are chromosomally unstable malignancies characterised by widespread genomic structural variation and copy number aberrations (Bowtell et al., 2015; Wang et al., 2017). Aside from mutations in *TP53* and *BRCA1*/*BRCA2*, driver mutations in other tumour suppressor or oncogenes are less common (Figure 1) (Kurman and Shih, 2016). Instead, structural change through DNA gains and losses are the main mechanisms for the inactivation of tumour suppressor genes (Wang et al., 2017). Pathogenic *TP53* mutations were identified in 96.7% of HGSOC cases and are believed to be an early mutational event essential for pathogenesis (Ahmed et al., 2010; Bowtell, 2010). Roughly 50% of HGSOC have defects in DNA repair and are a result of somatic/germline mutations and/or epigenetic silencing *via* methylation of HR related genes (Bowtell, 2010; Bowtell et al., 2015).

Mutations in *BRCA1/2* account for 10%–18% of hereditary OC cases, with somatic mutations in *BRCA1/2* accounting for another ∼4% of cases (Alsop et al., 2012; Cunningham et al., 2014). *BRCA1* and *BRCA2* are essential components of the HR pathway and are required for the repair of DNA double-stranded breaks (DSB) in DNA (Bowtell, 2010). In normal tissue, loss of BRCA1/2 triggers an apoptotic response involving p53. However, in HGSOC with loss or dysfunction of p53 as an initiating event, *BRCA1/2* loss leads to chromosomal instability and widespread copy number changes (Bowtell, 2010). G (Bowtell, 2010). Apart from *BRCA1/2* mutations, hypermethylation of the *BRCA1* or *RAD51C* gene promoters resulting in gene silencing is the next most common event, occurring in another 14% of cases (11% and 3% of cases respectively) (Network, 2011; Nesic et al., 2018). The remaining HRD HGSOC can be attributed to alterations in the Fanconi Anemia genes and other genes also involved in genome stability and DNA damage repair (*RAD51C, RAD51D, PALB2, ATM, ATR,* and *EMSY*) (Network, 2011). Loss of HR pathways requires tumours to rely on alternative, low fidelity mechanisms to repair DNA damage (Strickland et al., 2016). These error-prone pathways accumulate point mutations and random insertions/deletions resulting in the increase in mutational load, of potential relevance for immune therapies, as well as a distinct mutational signature (Patch et al., 2015; Strickland et al., 2016). This HRD signature has also been observed in carcinomas without known mutations in *BRCA1/2* or other HR genes, so it is possible there are more HRD HGSOC than currently hypothesised (Strickland et al., 2016). This HRD cohort likely contributes to the sensitivity of HGSOC to platinum and other DNA damaging agents, with an improvement of PFS and OS for HRD HGSOC compared to HR proficient cohorts.

Gene expression analysis has allowed the identification and validation of four subtypes of HGSOC: C1 (mesenchymal), C2 (immunoreactive), C4 (differentiated), and C5 (proliferative) (Tothill et al., 2008; Leong et al., 2015). Each subtype has distinct patterns of gene expression and clinical outcomes (Tothill et al., 2008). The C1 subtype displays a mesenchymal gene expression signature, extensive myofibroblast infiltration and poor survival rates (Tothill et al., 2008; Leong et al., 2015). In contrast, the C2 subtype is characterized by the presence of tumour infiltrating lymphocytes (TILs) and a more favourable prognosis (Zhang et al., 2003). Similarly, the presence of TILs has been noted in the C4 differentiated subtype along with a low stromal response and high expression of *MUC16/CA125* and *MUC1* (Tothill et al., 2008; Network, 2011). Thus, compared with C1 and C5 subtypes, C2 and C4 subtypes have a better prognosis, and may benefit from the use of immunotherapies in combination with chemotherapy or other DNA damaging agents such as PARPi (Tothill et al., 2008; Network, 2011)*.* The C5 subtype is driven by the suppression of the *Let7* family of microRNAs, leading to the amplification of stem-cell associated factors *MYCN* and *LIN28B, and the* low expression of differentiation markers including *MUC-16/CA-125* and other immune cell markers (Tothill et al., 2008; Helland et al., 2011; Leong et al., 2015). C1, C2 and C4 HGSOC displayed multiple subtype signatures, with most samples having a dominant signature (Zhang et al., 2014). The C5 subtype did not display a more dominant subtype, attributed to its stem-cell like, de-differentiated state (Zhang et al., 2014; Leong et al., 2015).

HRD positive and *BRCA*-mutant HGSOC have improved prognoses compared with their HR proficient counterparts, especially *BRCA2-*mutant HGSOC (Yang et al., 2011; Bolton et al., 2012; Strickland et al., 2016). This has been attributed to increased platinum sensitivity, however, the increased immunogenicity of these tumours could be an important contributing factor. A robust anti-tumour immune response relies on a cascade of interactions from the presentation of tumour-specific antigens, activation and trafficking of cytotoxic lymphocytes and the recognition and killing of tumour cells (Dunn et al., 2004a; Dunn et al., 2004b; Li et al., 2019). Specifically, the *BRCA1/2* mutant subset of HGSOC are associated with higher neoantigen loads, elevated levels of tumour infiltrating lymphocytes (TILs), and increased expression of immune pathway genes (Strickland et al., 2016).

Different lymphocyte subsets present in the tumour microenvironment (TME) can affect prognosis and tumour progression (Hendry et al., 2017). Most notably for HGSOC, the presence of CD8^+^ T cells, CD3^+^ T cells, and CD20^+^ B cells positively correlates with an improved overall survival (Hwang et al., 2012; Preston et al., 2013; Nelson, 2015; Hendry et al., 2017). Particularly, a higher ratio of CD8^+^ T cells to CD4^+^ CD25^+^ FOXP3^+/−^ regulatory T cells (Tregs) is associated with a better prognosis (Barnett et al., 2010; Preston et al., 2013). The mechanisms that define TIL attraction to these tumours continue to be studied but part could be attributed to the generation of tumour-specific antigens or neoantigens (Patch et al., 2015). Neoantigens are a class of human leukocyte antigen (HLA)-bound peptides that arise from tumour-specific mutations that elicit anti-tumour T-cell responses (Brown et al., 2014; Ott et al., 2017). HR-deficient HGSOC have a significant increase in neoantigen load compared to their HR proficient counterparts, correlating with the elevated level of TILs observed in HR-deficient carcinomas (Patch et al., 2015; Strickland et al., 2016). An additional mechanism of TIL attraction can be attributed to the activation of cyclic GMP-AMP synthase (cGAS) and STING pathways. The chromosomal instability that arises from BRCA1/2 loss in HGSOC lends to an increase in cytosolic DNA (ctDNA) fragments that bind to and stimulate the DNA-sensing cGAS/STING pathways and subsequently activates interferon (IFN) responses (Härtlova et al., 2015; Harding et al., 2017; Heijink et al., 2019; Reisländer et al., 2019). These pathways are an important part of the innate immune response and critical for dendritic cell (DC) activation and subsequent T cell priming against tumour cells (Flood et al., 2019).

A recent study demonstrated that *BRCA1* mutant HGSOC are prone to maintaining an obligatory inflammatory state through the upregulation of cGAS/STING signalling and producing an abundance of ctDNA fragments (Bruand et al., 2021). The loss of BRCA1 facilitated the enrichment of enhancers and the transcriptional upregulation of key genes in inflammatory pathways, DNA sensing pathways and IFN responses, committing tumour cells to an inflammatory state that promotes TIL recruitment (Bruand et al., 2021). To combat these immune responses, *BRCA1/2* mutant cells commonly downregulated CCL5 which significantly reduced T cell infiltration and attenuated inflammatory responses. This was supported by the prevalence of HGSOC with a methylated CCL5 locus lacking CD8^+^ TILs (Dangaj et al., 2019). Additionally, deletions of NFKB1 and IFNB1 alongside CCL5 were the most common in HRD HGSOC lacking immune activation and signalling (Bruand et al., 2021). Other immune evasion mechanisms included the upregulation of immune checkpoints such as programmed cell death-1 (PD-1) and the overexpression of vascular endothelial growth factor A (VEGF-A) an inducer of tumour angiogenesis, commonly seen in *BRCA1/2*-mutant HGSOC (Ruscito et al., 2018; Bruand et al., 2021). These evasion mechanisms present potential targets for treatment, as inhibiting these mechanisms may invigorate anti-tumour immune responses for more effective tumour clearance.

## 3 PARP inhibitor therapy in HGSOC

### 3.1 The PARP family and PARP inhibitors

PARPs are a family of proteins that are essential for several cellular processes including DNA repair, replication fork stability and genomic stability (Schreiber et al., 2006; Lord and Ashworth, 2017; Forment and O’Connor, 2018). PARP-1 and 2 act as DNA damage sensors, rapidly binding to breaks in DNA strands to hydrolyse NAD^+^ and produce linear and branched PAR chains in a process called poly(ADP-ribosyl)ation (PARylation) (Krishnakumar and Kraus, 2010; Rouleau et al., 2010; Murai et al., 2012). PARylation of chromatin proteins recruits DNA repair proteins to sites of damage causing PARP-1/2 to then dissociate from DNA *via* auto-PARylation (El‐Khamisy et al., 2003; Schreiber et al., 2006; Murai et al., 2012). The PARylation by PARP is not only important in DNA repair but chromatin modulation, regulation of DNA transcription and replication, protein degradation and cell cycle (Schreiber et al., 2006; Martí et al., 2020).

PARPi bind to the catalytic domains of PARP-1/2 and compete with NAD^+^, inhibiting PARylation, effectively disrupting recruitment of DNA repair proteins and PARP dissociation, thereby “trapping” PARP-1/2 on damaged DNA, and further reviewed in (D’Andrea, 2018; Wakefield et al., 2019). The trapping of PARP proteins on DNA stalls replication forks leading them to become dysregulated and collapse (Murai et al., 2012; Wakefield et al., 2019). Active PARP-1 regulates replication fork progression and when inhibited, replication fork stalling leads to a majority of single-stranded breaks (SSBs) being processed into double-stranded breaks (DSBs) (Berti et al., 2013). In healthy cells, these DSBs are repaired by the high-fidelity homologous recombination (HR) DNA repair pathway to successfully repair the damage (Ter Brugge et al., 2016).The HR DNA repair pathway is pivotal in accurate repair of DSBs and restarting stalled/collapsed replication forks, with BRCA1 and BRCA2 being crucial for the protection of replication forks during replication stress (Chen et al., 2018). In HGSOC cells with mutant *BRCA1/2* or other defective HR genes, the inhibition of PARP forces cancer cells to rely on error-prone repair DNA pathways or otherwise unrepaired damage persist into mitosis, leading to the rapid accumulation of mutations, genomic instability, and eventual cell death. The dual loss of the HR pathway and PARP function is synthetically lethal, in that the simultaneous inhibition of the two pathways leads to cell death, whereas loss of only one does not (Ashworth and Lord, 2018). It is within this realm of synthetic lethality that PARPi works best, as seen in the treatment of women with *BRCA*-mutant HGSOC experiencing sustained and profound responses to PARPi, compared with women with HR proficient carcinomas.

### 3.2 PARPi mechanisms of action

There are currently several PARPi available including olaparib, rucaparib, niraparib, veliparib, pamiparib and talazoparib being tested in phase III trials, with the first three mentioned having both Food and Drug Administration (FDA) and European Medicines Agency (EMA) approval for use in OC in the clinic (Pilié et al., 2019; Lee and Matulonis, 2020). A key feature of all PARPi molecules is a benzamide moiety that binds to the catalytic center of PARP, disrupting enzymatic activity. However, the disruption of catalytic activity alone is not enough to explain the vastly different outcomes in anti-tumour responses and efficacy in the clinic (Sun et al., 2018; Kim et al., 2021). The most effective PARPi trap PARP at sites of DNA damage and this could be due to difference in size and flexibility of each molecule influencing how PARPi bind and effect conformational changes. Allosteric destabilization of a critical helical regulatory domain neighboring the catalytic domain was crucial for cytotoxic and PARP-trapping effects with this being most prominent for rucaparib, niraparib and veliparib compared with olaparib and talazoparib (Zandarashvili et al., 2020). Talazoparib was reported to trap PARP roughly 100-fold more than niraparib, olaparib and rucaparib (Murai et al., 2014). However, the capacity for PARPi trapping does not relate to overall clinical benefit, as talazoparib is also noted for having increased toxicity in the clinic (Murai et al., 2014).

Additionally, another aspect of PARPi to consider is substrate selectivity and specificity. Most PARPi are highly selective toward PARP-1/2 although, computational in-silico analyses have uncovered 58 potential interactions with kinases of which only 10 were previously known (Thorsell et al., 2017; Antolin et al., 2020). Supporting this is evidence of rucaparib inhibiting the activity of kinases CDK16, PIM3 and DYRK1B in catalytic inhibition assays and additionally niraparib inhibiting the activity of two others, DYRK1A and DYRK1B (Antolin et al., 2020). Additional research in deciphering the specific mechanisms unique to each PARPi may elucidate novel pathways for clinical benefit. Investigation into improving PARPi specificity, tolerability and pharmacokinetic properties continues, with several PARPi in phase I/II clinical trials; including senaparib, which is 20-fold more potent than olaparib, and the highly selective, PARP-1 specific, PARP-1-DNA-trapper, AZD5305 (Cao, 2019; Johannes et al., 2021).

### 3.3 PARPi as a monotherapy in HGSOC

The PARPi olaparib and niraparib have been approved by both the EMA and FDA for use as maintenance therapy after response to first-line treatment with chemotherapy for women with germline or somatic *BRCA1/2* mutations or platinum-sensitive HGSOC respectively (Veneris et al., 2020). Additionally, olaparib, rucaparib, and niraparib are approved for use as a maintenance treatment for recurrent platinum-sensitive HGSOC patients and in some additional recurrent OC settings.

The phase III SOLO-1 trial evaluated the efficacy of olaparib in women with advanced *BRCA*-mutant platinum-sensitive HGSOC and demonstrated a 67% decrease in risk of disease progression or death (hazard ratio [HR] 0.33; 95% confidence interval [CI]: 0.25–0.43). Strikingly, at 5-year of follow-up, the PFS for the placebo arm was 13.8 months compared with the olaparib arm, on which women had achieved an unprecedented 56.0 months PFS, a 4-fold improvement, and 48% of women on olaparib remained disease free at this time, compared with only 20.5% of women on the placebo arm (Banerjee et al., 2021). Similarly, the phase III PRIMA/ENGOT-OV26/GOG-3012 trial (NCT026555016) examined responses to niraparib in platinum-sensitive advanced HGSOC and high grade endometrioid OC, regardless of *BRCA* mutation and/or HRD status (González-Martín et al., 2019). A significant improvement in PFS on niraparib maintenance was observed in the overall population with a median PFS of 13.8 months compared with 8.2 months for the placebo (HR 0.62, CI 0.50–0.76, *p* < 0.001). Roughly 50% of women were classified as having HGSOC with HRD and the greatest benefit derived from niraparib was seen in the subset of these with *BRCA-*mutations (median PFS 22.1 versus 10.9 months, HR 0.40, CI 0.27–0.62); followed by that observed in the non-*BRCA* HRD HGSOC subset (19.6 versus 8.2 months, HR 0.50, CI 0.31–0.83); lastly the remaining ∼50% of women had HGSOC which was HR proficient and responded the least well to PARPi (8.1 versus 5.4 months, HR 0.68, CI 0.49–0.94) (González-Martín et al., 2019). As seen in both clinical trials, response rates in women with HR proficient and platinum-resistant HGSOC were modest in comparison to HRD and platinum-sensitive HGSOC, thus a spectrum to the benefits derived from PARPi was observed. The combination of PARPi with other drugs to induce HRD in HR proficient disease or targeting other pathways that PARP-deficient tumours rely on, may be the answer to improving response further in HGSOC.

### 3.4 PARPi resistance

Regardless of the efficacy of PARPi as a monotherapy, a growing concern is the development of resistance with the prolonged use of PARPi. There are five main classes of resistance that have been characterised; drug efflux, changes in PAR metabolism, mutational changes of binding sites or target proteins, rewiring of stalled fork replication and restoration of the HR pathways (Wakefield et al., 2019). Several articles have reviewed these mechanisms in detail (Noordermeer and van Attikum, 2019; Wakefield et al., 2019; Kubalanza and Konecny, 2020), however the relevance of the different resistance mechanisms will need to be studied in large clinical cohorts for a better understanding of the selective pressures from PARPi in tumour evolution. The resistance landscape in patients is likely more diverse than what has been observed in research settings to date, thus developing a better understanding of the diversity could better inform therapeutic strategies moving forward. New technologies, including in proteomics (e.g., mass spectrometry and protein array analysis), that allow for the dissection of underlying molecular signaling events, could reveal clinically relevant biomarkers and new therapeutic choices for HGSOC, especially in the setting of the prediction and analysis of acquired PARPi resistance (Ghose et al., 2022). However, with our current knowledge, instigating early treatment with PARPi, rapid retreatment upon relapse and use of PARPi in combination therapies are important tools in maximizing PARPi efficacy. Treating early in the upfront maintenance setting, having first performed molecular analysis during first-line chemotherapy in order to match the HGSOC to appropriate combination PARPi therapy, may yield the most success in the treatment of highly heterogenous HGSOC.

## 4 PARP inhibitor effects beyond DNA repair

Studies of PARPi initially focused on DNA damage repair and *BRCA1/2* mutations. However, since then, the field of PARPi has expanded to include the roles PARP-1 has in chromatin structure, gene expression, and innate and adaptive immune responses.

### 4.1 Function of PARP-1 in chromatin remodeling and DNA methylation

In a normal state, DNA is wound around histones and non-histone proteins to form highly compact structures known as chromatin. When access to DNA is required, chromatin structures relax, unravelling bound DNA to allow protein complexes to bind and function, this reorganization of bound DNA is called chromatin remodelling (Sinha et al., 2021). Chromatin remodelling is important for maintaining genomic stability and is important in processes like DNA transcription, replication, and repair. PARP-1 plays an important role in these processes, by regulating chromatin remodelling *via* PARylation of the histones. In its latent state, PARP-1 is found bound to linker DNA and/or histone proteins, resulting in the condensed structure of chromatin called heterochromatin. In this state, no transcription machinery can access the DNA, repressing gene transcription. In the presence of DNA damage however, PARP-1 becomes active (Kim et al., 2004; Muthurajan et al., 2014). Active PARP-1 PARylates itself and histones, promoting the remodelling of chromatin to become euchromatin as the addition of negatively charged PARs on the histones repels DNA. At sites of DNA damage, histone PARylation causes its eviction from DNA strands, facilitating the recruitment of other chromatin remodelers and further loosening of chromatin for subsequent recruitment of DNA repair proteins (Quénet et al., 2009). More specifically, PARP-1 PARylates and then binds to chromatin remodelers at their PAR-binding domain for subsequent alteration of chromatin structures (Andronikou and Rottenberg, 2021). For example, PARylation of the lysine specific demethylase 4D (KDM4D) at its C-terminal promotes demethylation of the methylated forms of H3K9, reducing chromatin compaction, and allowing gene transcription (Khoury-Haddad et al. 2014). However, PARylation of KDM4D at its N-terminal inhibits the action of this enzyme at the promoter of retinoic acid receptor-dependent genes and represses gene transcription (Le May et al., 2012). In this case, the use of PARPi may abolish this specific PARP activity in chromatin remodelling machinery. Particularly, PARPi interferes with the recruitment of KDM4D to double stranded breaks and thus inhibits the repair process (Khoury-Haddad et al. 2014).

There are several natural inhibitors that can counteract PARP-1’s involvement in chromatin remodelling machinery, one of them is poly-ADP-ribose glycohydrolase (PARG). PARG counteracts the action of PARP-1 by cleaving the PAR on PARylated PARP-1, rendering it inactive (Kim et al., 2004). Amplified in liver cancer protein 1 (ALC1) is a chromatin remodeler that is rapidly recruited to DNA-damage and binds to PARylated PARP-1 (Pines et al., 2012; Ahel et al., 2009). When ALC1 binds to PARylated PARP-1, it not only activates the protein but secondarily protects PAR on PARP-1 from PARG hydrolysis (Ahel et al., 2009; Gottschalk et al., 2012; Singh et al., 2017). Loss of ALC1, and subsequent loss of PAR protection by ALC1, was found to enhance PARP-1/2 trapping on DNA by PARPi, effectively sensitising cells to PARPi (Blessing et al., 2020; Juhász et al., 2020).

Another natural inhibitor of PARP-1 activity is macroH2A1.1 which binds to autoPARylated PARP-1 to prevent PAR hydrolysis, which can promote chromatin recondensation to interfere with transcriptional processes (Timinszky et al., 2009). MacroH2A1.1 is a splice variant of macroH2A1, which is recruited to DSBs and is implicated in regulating PAR metabolism and NAD + turnover (Ruiz et al., 2019). The alternative splice variant, macroH2A1.2, interacts with other enzymes to recondense chromatin through the production of H3K9 methylation marks (Khurana et al., 2014; Alagoz et al., 2015). These compact chromatin marks attract BRCA1, promoting the use of the HR pathway to repair DNA damage (Lee et al., 2013; Alagoz et al., 2015). Loss of macroH2A1.1 has been noted in several cancers and, due to its roles in chromatin condensation and BRCA1 recruitment, depletion of this histone may increase PARPi sensitivity (Ruiz et al., 2019).

DNA methylation is another major epigenetic modification which occurs at the fifth carbon of cytosine when followed by guanine (CpG) in eukaryotic genomes. The methylated cytosine (5 mC) is induced and maintained by DNA methyltransferase (DNMT). Promoter hypermethylation commonly promotes gene silencing. This epigenetic silencing has been observed in HR genes, including *BRCA1* or *RAD51C*, occurring as an early clonal event, contributing to the development of OC cases (Alsop et al., 2012) *BRCA1* can in fact partially predict BRCAness in OC (Aref-Eshghi et al., 2020). Homozygous methylation (of all copies present) of the *BRCA1* promoter can predict sensitivity to PARPi therapy. On the other hand, heterozygous methylation, (loss of methylation of any copy of the gene present in the cancer), correlates with PARPi resistance (Kondrashova et al., 2018). Similarly, for *RAD51C,* complete gene silencing correlates with PARPi response whilst loss of methylation of even one allele of *RAD51C* drives resistance to PARPi (Nesic et al., 2021).

PARP-1 interaction with DNMT1 contributes to the regulation of DNA methylation. PARylation has been shown to maintain unmethylated CpG at specific sites of the genome, while blockade of PARylation increases DNA methylation levels in the genome (DE CAPOA et al., 1999; Zampieri et al., 2012). Interestingly, the modulation of DNA methylation by PARP-1 can be counteracted by CCCTC-binding factor (CTCF), that can induce the auto-modification of PARP-1 (Guastafierro et al., 2008; Zampieri et al., 2012). Pharmacological inhibition of PARP-1 has been shown to change the genome-wide DNA methylation profile, confirming PARP involvement in DNA methylation processes (Nalabothula et al., 2015). Besides inducing more DNA methylation, PARPi has been shown to induce the expression of enhancer of zeste homolog 2 (EZH2), a histone methyltransferase that catalyses trimethylation of the lysine residue on histone3 (Martin et al., 2015). This in turns results in a genome-wide increase of H3K27me3 and thus chromatin compaction (Martin et al., 2015). Both increases in DNA methylation and H3K27me3 in the genome result in increased heterochromatin structure and further silencing of various genes.

#### 4.1.1 Exploiting the epigenome with PARPi

In BRCA1/2 defective cancer cells, when the backup DNA repair pathways are disrupted, PARPi can induce synthetic lethality. Thus, inducing the complete loss of DNA repair capability has been strategized to kill BRCA-proficient cancer cells, by combining PARPi with epigenetic drugs that can induce a BRCA defective-like phenotype (DE CAPOA et al., 1999; Caiafa et al., 2009; Abbotts et al., 2019). Combining PARPi with epigenetic drugs can also sensitize PARPi-resistant cancer cells, thus overcoming resistance to treatment (DE CAPOA et al., 1999; Abbotts et al., 2019; Caiafa et al., 2009; Cimmino et al., 2017; Eckschlager et al, 2017). Several epigenetic-targeting drugs have been suggested for use in combination therapy with PARPi for not only BRCA-defective cancers, but also BRCA-proficient cancers, for which therapy choices are more limited (Abbotts et al., 2019; Cimmino et al., 2017; Eckschlager et al, 2017).

Several studies have demonstrated the use of low doses of DNMT inhibitors (DNMTi) in combination with PARPi to target HR pathways in BRCA-proficient triple-negative breast cancer, ovarian cancer, acute myeloid leukemia, and non-small cell lung cancer (Muvarak et al., 2016; Pulliam et al., 2018; Abbotts et al., 2019). DNMTi are cytidine analogs that, following their incorporation into DNA, covalently entrap the methylation maintenance enzyme, DNMT1 (Santi et al., 1984). Several DNMTi are currently in clinical trials, and two of them, decitabine and 5-azacytidine, have been approved for the treatment of myelodysplastic syndrome and acute myeloid leukemia (Hu et al., 2021). PARP-1 is in fact crucial for DNMT1 to function properly by protecting the *DNMT1* promoter from being methylated, and also by non-covalently interacting with DNMT1 to promote its methylating activity (Reale et al., 2005; Caiafa et al., 2009; Zampieri et al., 2009). A combination of PARPi and DNMTi has been shown to promote cytotoxicity, as DNMTi creates a BRCA-defective-like phenotype through repression of HR and nonhomologous end-joining (NHEJ) genes, while PARPi inhibits HR and thus enhances DNMTi functionality (Abbotts et al., 2019). Additionally, Muvarak et al. (2016) shows that a combination of a DNMTi, 5-azacytidine, and PARPi, talazoparib, increased the trapping time of PARP at DNA damage sites from 30 min to up to six hours, preventing PARP from fixing DNA damage for a longer period, providing a potential therapeutic strategy.

A genome-wide RNAi screen by Kharat et al. (2020) associated loss of TET2 with the development of resistance to PARPi. Depletion of TET2 reduces the conversion of DNA methylation mark 5-methylcytosine, to 5-hydroxymethycytosine (5 hmC), the first step in the demethylation process. Subsequently, replication forks in cancer cells fail to degrade and in turn, this promotes resistance to PARPi in cancer cells. When cells were treated chemically to increase 5 hmC abundance, the replications forks were degraded by the recruited base excision repair-associated apurinic/apyrimidinic endonuclease (APE1), independent of BRCA status (Kharat et al., 2020). These findings suggest that exposure to epigenetic drugs that induce TET2 activity or increase 5 hmC abundance may induce PARPi sensitivity. Indeed, Sajadian et al. (2015) showed that the active demethylation by anti-cancer DNMTi, 5-azacytidine, is TET2 dependent, while Cimmino et al. (2017)restored sensitivity of TET2-deficient cancer cells to PARPi by increasing the abundance of 5 hmC using ascorbic acid.

Another type of epigenetic drug that could augment the effect of PARPi is the histone deacetylase inhibitors (HDACi). HDAC removes acetyl groups from the lysine residues of histone tails and has been shown to play various roles in cancer initiation, progression, metastasis and angiogenesis, thus it has emerged as anticancer drug (Eckschlager et al, 2017). Several HDACi have entered clinical trials, and four have been approved by the FDA. These include vorinostat, romidepsin and belinostat for T-cell lymphoma and panobinostat for multiple myeloma (Eckschlager et al, 2017). In prostate cancer, HDAC inhibition by HDACi results in downregulation of HR DNA repair genes by reduction of the recruitment of the activating transcription factor, E2F1 to the promoter of these genes (Kachhap et al., 2010). Several *in vitro* studies show an augmented efficacy of PARPi at targeting HR pathways when combined with HDACi (Adimoolam et al., 2007; Ha et al., 2014; Liu et al., 2015). Here, the HDACi induces a BRCA defective-like phenotype, by depleting the expression and reducing the recruitment of HR proteins thus increasing the sensitivity of the cancer cells towards PARPi (Ha et al., 2014; Liu et al., 2015). Since a monotherapy with HDACi alone has not resulted in an effective treatment, a combination of HDACi with PARPi therapy is now under investigation for OC in the clinic (NCT03924245) (Mackay et al., 2010). The induction of BRCAness by HDACi may allow the combination therapy to effectively treat OC, independent of their BRCA status.

## 5 Immuno-modulatory effects of PARPi

### 5.1 Extra-tumoural effects of PARPi in immune cell subsets

With important roles in DNA regulation, PARP-1/2 play a role in T-cell development, differentiation, and function. The development of T-cells is a complex and highly regulated process that begins in the thymus with bone marrow-derived lymphoid precursors and through well-characterized maturation steps give rise to mature T-cells (Koch and Radtke, 2011). PARP-1 modulates activity of nuclear factor of activated T-cells (NFAT) which drives CD4^+^ T-cell differentiation (Olabisi et al., 2008; Valdor et al., 2008). A reduction in the expression of NFAT reliant cytokines was observed in PARP-1 deficient T-cells and furthermore PARP-1 deficiency creates a bias for CD4^+^ T-cell differentiation to a Th1 phenotype (Macian, 2005; Olabisi et al., 2008; Sambucci et al., 2013). The Th1 subset of CD4^+^ T-cells is associated with the production of cytokines such as IFN- γ, IL-2 and TNF -β that induce inflammation and cell-mediated immune responses (Constant and Bottomly, 1997). The Th2 subset promotes B-cell proliferation and differentiation through IL-4 and IL-5 cytokine production and is associated with humoral-type immune responses (Constant and Bottomly, 1997). There is conflicting data on PARP-1 deficiency driving Th1 differentiation of CD4^+^ T-cells, with one study in a model of airway inflammation observing olaparib promoting the Th1 phenotype whereas a model of inflammatory arthritis observed PARP inhibition associated with a suppression of Th-1-associated cytokines. Thus, PARP driven Th1 differentiation is likely mediated by other context-specific factors.

During early T-cell development, PARP2 is essential for the development of CD4/CD8 double positive thymocytes and PARP-1 regulates expression of Foxp3 in CD4^+^ T-cells (Zhang et al., 2013; Luo et al., 2015; Navarro et al., 2017). *In-vitro* studies show that PARP-1 and PARP2 deficient T cells have a decrease in total CD8^+^ and CD4^+^ populations (Navarro et al., 2017). This observation was prevalent in singular deficiencies and with the dual loss of both PARP-1 and PARP2, with the dual loss having a more dramatic reduction suggesting a, and there was prevalent amounts of DNA damage detected suggesting the reduction in these T-cell populations are a result of accumulating DNA damage and genomic instability and not entirely a block in maturation (Navarro et al., 2017). In the circumstance of PARP-1 deficiency, populations of CD4^+^ T-cells expressing Foxp3 increases due to the lack of Foxp3 PARylation for subsequent degradation (Luo et al., 2015). Expression of Foxp3 on CD4^+^ T-cells causes differentiation into Tregs which are immunosuppressive through their production of inhibitory cytokines, mediation of cytolysis and modulation of DC maturation or function (Vignali et al., 2008; Zhang et al., 2013; Luo et al., 2015). *In vivo* models studying olaparib in BRCA1-deficient ovarian cancer observed significantly increased proportions of CD4^+^ and CD8^+^ effector T-cells infiltrating intratumorally and peripherally and, notably, an increase in intra-tumoral CD4/Foxp3+ Tregs was not seen (Ding et al., 2018). Suggesting that treatment with PARPi in an *in vivo* setting does not disrupt T-cell development and function to the extent of tumour benefit. Additionally, olaparib-treated CD8^+^ T-cells showed reduced expression of immune receptors, such as PD-L1, that are associated with T-cell inhibition and exhaustion and produced higher levels of TNFα and IFNγ (Ding et al., 2018).

The recruitment of DC to the tumour microenvironment is an important step in the anti-tumour immune response as they play roles in activating and inducing the differentiation of T-cells (Patente et al., 2019). There is evidence PARPi has an indirect effect in activating DC though its DNA-damaging abilities and creation of cytosolic DNA fragments. Cytosolic DNA activates the cGAS/STING pathway within the cell but can also be exocytosed to activate STING pathways in neighbouring DC (Mouw et al., 2017; Pantelidou et al., 2019). The activation of cGAS/STING was noted in a PARPi treated BRCA1-deficient mouse model of TNBC, but not in DC treated with PARPi alone (Pantelidou et al., 2019). This suggests that DC cGAS/STING activation is not induced by PARPi alone. This notion was supported in a BRCA1 deficient model of OC, with activation of cGAS/STING observed upon treatment with olaparib (Ding et al., 2018). To confirm the paracrine effect of PARPi on DC activation, PARPi treated ovarian cells were co-cultured with naïve DC. Increased levels of TBK-1, IRF3, CXCL10 and IFNβ were observed, confirming cGAS/STING activation and expression of downstream genes, further supporting the indirect activation of DC upon treatment with PARPi (Ding et al., 2018). Furthermore, treatment with PARPi increased DC populations with increased antigen presentation machinery, specifically upregulated costimulatory CD80 and CD86 and antigen presenting major histocompatibility complex class II (MHC II) (Ding et al., 2018; Pantelidou et al., 2019).

Natural killer (NK) cells are effector lymphocytes utilized in the innate immune response against “non-self” cells and “self” cells undergoing stress in the form of infections or malignant transformations (Vivier et al., 2004). When activated, NK cells either have direct cytotoxic attacks on targets or produce large arrays of cytokines and chemokines to initiate antigen-specific immune responses. Specifically, NK cells can directly interact with cells through TNF-related apoptosis-inducing ligand (TRAIL) and the Fas ligand to induce apoptosis or indirectly through secretion of IFNγ and TNFα (Barrow and Colonna, 2017; Souza-Fonseca-Guimaraes et al., 2019). In tumour cells, TRAIL stimulates PARP-1 activation and subsequently the PARylation of high-mobility group box protein 1 (HMGB1) which results in HMGB1 localisation to the cytoplasm. This localisation from nucleus to cytoplasm promotes an autophagic response and protects the tumour cells from TRAIL mediated apoptosis (Yang et al., 2015). Treatment with PARPi suppressed the PARP-1/HMGB1 pathway and re-sensitized tumour cells to TRAIL induced cell death suggesting PARPi can sensitize tumours to NK-cell mediated apoptosis (Yang et al., 2015). Treatment with PARPi has also been shown to upregulate death receptors Fas and death receptor 5 in several cancer cell lines (Meng et al., 2014). Upregulation of these receptors sensitized cells to TRAIL induced apoptosis. This was further supported by a study observing NK cell killing in prostate cancer cells with and without PARPi treatment independent of BRCA status (Fenerty et al., 2018). They found treating tumour cells with olaparib upregulated death receptor TRAIL-2 and significantly increase tumour cell sensitivity to NK cell killing in both BRCA-wildtype and BRCA-mutant cells. Additionally, they replicated these results in additional tumour cell lines, including breast, chordoma, non-small cell lung carcinoma (Fenerty et al., 2018). It is important to note that the presence of NK cells has a favourable impact on OS for HGSOC patients and these findings suggest that PARPi can recruit and sensitize tumour cells to NK cells, and that NK cells contribute to PARPi anti-tumour effects (Henriksen et al., 2020).

### 5.2 Intra-tumoural effects of PARPi on inflammation, the cGAS-STING pathway, and immune checkpoint expression

Tumours with existing DNA repair defects initially stimulate inflammation and T_H_1 immune responses, however, maintaining a constant level of DNA damage and subsequent chronic inflammatory responses encourages the infiltration of immune-suppressive cells (Schreiber et al., 2011; Crusz and Balkwill, 2015; Fridman et al., 2017). Chronic inflammation promotes immunosuppression and in cancer this promotes tumour progression. However, studies suggest PARPi has the potential to counteract this and reinvigorate the anti-tumour immune response (Fridman et al., 2017).

Inflammatory responses can be promoted by PARP-1 through its regulation of several transcription factors, cytokines and chemokines (Pazzaglia and Pioli, 2020). Nuclear factor κB (NF-κB) is a transcription factor that is important in the regulation of genes for inflammatory, apoptotic and cell proliferative responses, and for complete NF-κB-dependent gene transcription PARP-1 acetylation is required (Hassa et al., 2005). Additionally, PARP-1 can activate NF-κB through several mechanisms including through the mono-ubiquitination of NF-κB essential modulator (NEMO) for NF-κB nuclear translocation and sustaining toll-like receptor (TLR) induced NF-κB activation (Stilmann et al., 2009; Hinz et al., 2010; Ji et al., 2016). To study whether treatment with PARPi affects inflammatory responses, Alvarado-Cruz et al. (2021) interrogated *BRCA1-*mutant triple negative breast cancer (TNBC) cell lines and tumours samples after treatment with veliparib for three weeks. Upregulation of hallmark inflammatory TNFα pathways were observed after treatment with PARPi specifically in the BRCA1-deficient cells, and the mechanistic basis for this upregulation was through the cGAS/STING pathways. Separately, a study using genetically engineered mouse models (GEMM) of HGSOC investigated the effects of olaparib in *BRCA1* deficient and *BRCA1* wildtype settings. Treatment with olaparib elicited an increase in CD4^+^ and CD8^+^ T-cells as well as a pronounced increase in IFNγ and TNFα (Ding et al., 2018). The increase in CD4^+^ and CD8^+^ T-cells was also associated with the increased presence of DC with a potent antigen presenting capacity, and a decrease in MDSCs in the tumour, spleen and blood (Ding et al., 2018). These responses seen were restricted to the *BRCA1* deficient GEMM and mechanistically were associated with the stimulation of the STING pathway. It was proposed that PARPi induced DSBs creating cytosolic DNA fragments that are bound by cGAS, activating STING, and subsequently the production of pro-inflammatory cytokines and IFN responses (Pantelidou et al., 2019; Shen et al., 2019). The recent study by Bruand et al.(2021) suggests that *BRCA* mutant cells are intrinsically programmed for the cGAS/STING/IFN signalling seen in HGSOC and that PARPi enhances this signalling by increasing the amount of ctDNA present. This potentially explains why the immune responses observed after treatment with PARPi were isolated to *BRCA1* deficient GEMM models.

Another study interrogated the role of STING in relation to PARPi in both *in vitro* and *in vivo* experiments regardless of *BRCA1/2* status (Shen et al., 2019). Treating OC cells with talazoparib markedly elevated the phosphorylation of two key components along the STING pathway, IRF3 and TBK1. An increase of total IRF3 and TBK1 translocation from the cytoplasm to the nucleus was observed, suggesting functional signaling of STING (Shen et al., 2019). Additionally, CCL5 and CXCL10, which are two major chemokines activated by STING that positively correlate with the presence of CD8^+^ T-cells, were seen to be upregulated post PARPi treatment. The knockdown of STING, TBK1, IRF3 or cGAS significantly reduced the upregulation of CCL5 and CXCL10 in PARPi treatment in OC cell lines. Further work in mouse models validated these findings showing treatment with PARPi elicited the expression of CCL5 and CXCL10 and induced higher percentages of CD8^+^ T-cells and PD-L1+ cells infiltrating the TME. Treatment with PARPi had no therapeutic effects in immunodeficient mice but prolonged survival and limited tumour growth in immune competent mice (Shen et al., 2019). Additionally, knockout of STING abolished the anti-tumour effects of PARPi establishing PARPi efficacy is based in an immunogenic response. These results do not correlate with the previous studies mentioned thus further work to determine the role of BRCA-loss in STING and IFN responses in HGSOC is needed, which will hopefully further elucidate mechanisms by which PARPi invigorates immune responses.

Both studies also noted the increased expression of programmed cell death-ligand 1 (PD-L1) in cells when treated with PARPi (Ding et al., 2018; Shen et al., 2019). Programmed death-ligand 1 is the ligand of PD-1, which is an immune receptor expressed on CD4^+^/CD8+ T-cells and B cells, and mediates the inhibition of T-cell proliferation and IFNγ production (Iwai et al., 2017). The role of PD-1/PD-L1 is to mediate autoimmune responses, however in cancer, the upregulation of immune checkpoints can be used to suppress the anti-tumour immune response. Upregulation of PD-L1 expression can occur through several mechanisms including PD-L1 promotor binding by NF-kB, JAK1/2 activation and IFNy secretion following type I IFN response (Bellucci et al., 2015; Chabanon et al., 2019; Cai et al., 2020). All of these effects can be stimulated through the cGAS/STING pathway that is activated upon treatment with PARPi. Shen et al., found treatment with PARPi increased percentages of PD-L1+ cells and explored the effects of combining talazoparib and an anti-PD-L1 antibody. Tumours treated with the combination therapy significantly reduced tumour burden compared to either monotherapy, and had the most significant increase in CD8^+^ cell recruitment (Shen et al., 2019). Additionally Jiao et al. (2017) demonstrated that PARPi induced PD-L1 expression regardless of BRCA-status, and effectively reduced the efficacy of active cytotoxic T-cells. Combination of olaparib and an anti-PD-L1 antibody desensitised PARPi treated cells and found the combination more effective than either agent alone (Jiao et al., 2017). Due to the encouraging results of PARPi and checkpoint inhibitor combinations in research, this combination is being explored in several clinical trials.

#### 5.2.1 Combining PARPi with immune checkpoint inhibitors

Currently, the most promising immunotherapy for HGSOC are immune checkpoint inhibitors (ICI), which are monoclonal antibodies that disrupt signalling that would promote effector T-cell deactivation. Most common ICI for HGSOC are antibodies that target PD-1 and Cytotoxic T-Lymphocyte Antigen 4 (CTLA-4), as a monotherapy they have had some efficacy in patients but few durable responses (Cortez et al., 2018; Färkkilä et al., 2020). The phase II KEYNOTE-100 clinical trial of pembrolizumab (an anti-PD-1) as a monotherapy in EOC had an objective response rate (ORR) of only 8% and median progression free survival (PFS) of 2.1 months (Matulonis et al., 2018). Similarly, results from the clinical trial JAVELIN in recurrent OC with avelumab (anti-PD-L1) yielded an ORR of 9.6% and median PFS of 2.6 months (Disis et al., 2019). This could be, in part, due to the low expression of PD-L1 on tumour cells or the highly immunosuppressive TME that develops during a prolonged immune response (Gashi, 2022). Offering ICI early in the treatment of EOC could improve poor responses rates by treating before an immunosuppressive TME develops, potentially delaying the development of immune evasion. Alternatively, the combination of ICI therapy with other therapeutics such as PARPi could mitigate issues of timing of delivery, by driving immunogenicity and reviving immune responses through accumulative DNA damage and tumour specific mutations.

In the phase I/II clinical trial TOPACIO/KEYNOTE-162 (NCT02657889) investigating the combination of niraparib/pembrolizumab (anti-PD-1) in recurrent OC and TNBC, an ORR of 25% was achieved in the general cohort which was an improvement compared to response rates of PARPi or IC as a monotherapy (Konstantinopoulos et al., 2018; Konstantinopoulos et al., 2019). Additionally, the study highlighted the importance of using predictive biomarkers to identify patients who will benefit most from this combination therapy and found that tumours with a mutational signature 3 (HRD signature) and a positive immune-score for exhausted CD8^+^ T cells were associated with an improved response (Färkkilä et al., 2020). The phase II MEDIOLA (NCT02734004) trial evaluating the combination of olaparib/durvalumab in recurrent platinum-sensitive EOC observed a median PFS of 11.1 months (95% CI: 8.2, 15.9) and ORR of 71.9% (95% CI: 53.25%, 86.25%) with a partial response (PR) rate of 53% (17/32) and complete response (CR) rate of 21.8% (7/32). To date, the median overall survival (OS) for all patients has not been reached, with 87% of patients alive at 24 months (Drew et al., 2019). The combination treatment was well tolerated with the most common adverse events (AE) being anaemia (17.6%), elevated lipase (11.8%), neutropenia (8.8%) and lymphopenia (8.8%), and only eight out of 32 patients discontinuing olaparib or durvalumab due to an AE. Currently there are several clinical trials ongoing, looking at PARPi and ICI in the frontline maintenance setting and the results should further define the benefits to be derived from this combination (Table 1). However, the response rates from available clinical trial data in platinum-sensitive HGSOC look promising and exploring the potential of ICI in other combinations may also be beneficial. There are other clinical trials investigating triple combinations of PARPi and ICI with other drugs commonly used in the treatment of HGSOC with varying success and these will be discussed below.

**TABLE 1:** Clinical trials combining PARP inhibitors with chemotherapy or with therapeutics with relevance for the immune system. This includes immune checkpoint inhibitors and/or angiogenesis agents in order to attempt to elicit more robust and durable responses.

Study and Phase	Drugs	Cohort	Outcomes	Side effects
TOPACIO/KEYNOTE-162 Phase I/II (NCT02657889)	Niraparib + Pembrolizumab	Recurrent platinum-resistant HGSOC	ORR 18%; DCR 68%; overall ORR 45%; DCR 73% in *BRCAmut* pts	Common grade ≥ 3 AEs; anemia (19%) and thrombocytopenia (9%)
MEDIOLA Phase II (NCT02734004)	Olaparib + Durvalumab	Recurrent platinum-sensitive EOC	28-wk DCR 65.6%; ORR 71.9%; mPFS 11.1 mos; mOS for all pts not yet reached	Common ≥ Grade 3 AEs; anaemia (17.6%), elevated lipase (11.8%), neutropenia (8.8%), and lymphopenia (8.8%).
Phase I/II (NCT02571725)	Olaparib + Tremelimumab	Recurrent *BRCA* mutant OC	Awaiting results	N/A
Phase I/II (NCT02953457)	Olaparib + Durvalumab + Tremelimumab	Recurrent platinum-sensitive/resistant/refractory EOC, Fallopian Tube, or Primary Peritoneal Cancer	Awaiting results	N/A
NSGO-AVANOVA2/ENGOT-ov24 Phase II	Niraparib + Bevacizumab	HGSOC or endometrioid platinum-sensitive recurrent OC	mPFS 11.9 mos; mPFS 14.4 mos in *BRCAmut* pts; mPFS 11.9 mos in HRD pts; mPFS 11.3 mos in non-*BRCAmut* pts;	Common ≥ Grade 3 AEs; anaemia (15%), thrombocytopenia (10%) and hypertension (21%)
PAOLA-1/ENGOT-ov25 Phase III (NCT02477644)	Olaparib + Bevacizumab	Recurrent HGSOC	mPFS 36.5 mos; mPFS 50.3 mos in HRD positive pts; mPFS 24.4 mos in HRD negative pts; mPFS 34.0 mos in HRD unknown pts	Common Grade ≥3 AEs; hypertension (19% ) and anaemia (17%). Five treatment emergent AEs of death (olaparib, *n* = 1; placebo, *n* = 4)
MITO25 Phase II (NCT03462212)	Rucaparib + Bevacizumab	HRD and HR HGSOC	Ongoing and awaiting results	N/A
Phase II trial	Olaparib + Cediranib	Recurrent platinum-sensitive, HGSOC or endometrioid OC	mPFS 16.5 mos; overall No significance in OS; mPFS 16.4 vs. 16.5 mos (control arm) in *BRCAmut* pts; mPFS 23.7 vs. 5.7mos (control arm) in *BRCA-*wildtype pts	Grade 3 and 4 AEs; fatigue (12 pts), diarrhoea (10 pts), and hypertension (18 pts)
NRG- GY004 phase III trial (NCT02446600)	Olaparib + Cediranib vs. Olaparib vs. Chemo	Recurrent platinum-sensitive HGSOC or high-grade endometrioid ovarian, primary peritoneal, or fallopian tube cancers.	mPFS 13.7 mos in HR-deficient pts; mPFS 8.3 mos in HR proficient pts; mPFS 20.4 vs. 12.3 vs. 13.1 mos (combo vs. chemo vs. PARPi) in HR-deficient pts	N/A
Single arm trial EVOLVE Phase II	Olaparib + Cediranib	PARPi-resistant HGSOC	16-week PFS rates 55% (platinum-sensitive after PARPi), 50% (platinum-resistant after PARPi), and 39% (exploratory cohort)	Grade 3 toxicities; diarrhea (12%) and anemia (9%)
ICON9 Phase III (NCT03278717)	Olaparib + Cediranib	Recurrent platinum-sensitive OC	Ongoing	N/A
COCOS Phase II/III (NCT02502266)	Olaparib + Cediranib	Recurrent Platinum-Resistant or -Refractory Ovarian, Fallopian Tube, or Primary Peritoneal Cancer	Ongoing	N/A
Phase II open-label study (NCT03574779)	Niraparib + Dostarlimab + Bevacizumab	Recurrent platinum-resistant EOC	mPFS 7.6 mos; DCR 76.9%	Common grade ≥3 TEAEs; hypertension (22.0%), fatigue (17.1%), and anemia (17.1%). Common serious TEAEs; thrombocytopenia (7.3%), anemia (4.9%), and hypertension (4.9%).
MEDIOLA Phase II (NCT02734004)	Olaparib + Durvalumab + Bevacizumab	Recurrent platinum-sensitive non-germline *BRCA* mutant OC	24-week DCR 77.4%; ORR 77.4%; mPFS 14.7	Common grade ≥ 3 AEs in O + D; anaemia, lipase increased and neutropenia and anaemia, hypertension, fatigue, lipase increased, and neutropenia
Phase I trial (NCT00516724)	Olaparib + Carboplatin +/or paclitaxel	Advanced solid tumours refractory to standard treatments	Increased hematologic toxicities, made establishing a dosing regimen difficult	Grade 1/2 DLTs; thrombocytopenia and neutropenia. Non-hematologic grade 1/2 AEs; fatigue (70%), nausea (40%), neutropenia (51%) , thrombocytopenia (25%)
VELIA/GOG-3005 Phase III	Veliparib + carboplatin-paclitaxel	Newly diagnosed HGSOC	mPFS 29.3 mos vs. 19.2 mos (veliparib combo vs. placebo)	Nausea and fatigue common in overall cohort. Veliparib combo had higher incidence of anemia and thrombocytopenia
Phase II clinical trial (NCT01306032)	Veliparib + Oral cyclophosphamide	Recurrent *BRCA*-mutant OC	mPFS 2.3 mos vs. 2.1 mos (combo vs. cyclophosphamide alone)	Common grade 2/3 AEs; leucopenia and lymphopenia
Phase II clinical trial (NCT02853318)	Pembrolizumab + Bevacizumab + Oral cyclophosphamide	Recurrent OC	ORR 47.5%; mPFS 10 mos	Common AEs fatigue; [18 (45.0%)], diarrhea [13 (32.5%)], and hypertension [11 (27.5%)].

ORR, overall respone rate; DCR, disease control rate; *BRCA*mut, *BRCA*mutant; mos, months; pts, patients; mPFS, median progression free survival; mOS, median overall survival; AEs, adverse events; TEAEs, treatment emergent adverse events; N/A, not available; HRD, homologous repair deficient; HGSOC, high grade serous ovarian carcinoma; EOC, epithelial ovarian cancer; OC, ovarian cancer; PARPi, PARP inhibitor

#### 5.2.2 Anti-angiogenics and PARPi

Bevacizumab (BV) is an anti-VEGFR antibody already in use for the treatment of HGSOC in combination with standard chemotherapies. It targets the cytokine VEGF-A which is secreted by tumour cells and binds to VEGFR-1 and VEGFR-2 receptors, promoting angiogenesis (the formation of blood vessels that allow for tumour growth) and metastasis. VEGF-A has been shown to be overexpressed in *BRCA1* mutant HGSOC and its inhibition increases hypoxia and subsequent downregulation of HR genes (specifically *BRCA1/2* and *RAD51C*) as a result (Bindra et al., 2004; Bindra et al., 2005; Ruscito et al., 2018; Bruand et al., 2021). This decrease in DNA-repair potential could sensitise tumours to PARPi, thus providing a rationale for combining PARPi plus BV therapy in clinical trials. Inhibition of VEGF has also been shown to reduce MDSC, Treg and TAM populations and increase T-cell activation and priming, enhance DC antigen presentation and encourage TIL presence (Goel et al., 2011; Hegde et al., 2018). It has been studied in combination with both olaparib and niraparib in phase I studies, with both showing tolerability without dose-limiting toxicities (Dean et al., 2012). The phase II clinical trial NSGO-AVANOVA2/ENGOT-ov24 studied BV and niraparib compared to niraparib monotherapy in platinum-sensitive recurrent HGSOC or endometrioid ovarian cancer. The combined treatment of BV and niraparib significantly improved patient outcomes compared to niraparib alone with a median PFS of 11.9 versus 5.5 months (HR: 0.35, CI 0.21–0.57, *p* < 0.001) (Mirza et al., 2019). The *BRCA*-mutant patient cohort derived the most benefit with a PFS of 14.4 versus 9.0 months, (HR 0.49, CI 0.21–1.15), followed by the HR-deficient subgroup with a PFS of 11.9 vs. 4.1 months (HR 0.19, CI 0.06–0.59 and then the non-*BRCA-*mutant patients with a PFS of 11.3 versus 4.2 months (HR 0.32, CI 0.17–0.58) (Mirza et al., 2019). Overall, patients on the combination of BV and PARPi did significantly better regardless of HR-status.

In phase III PAOLA-1 clinical trial (ENGOT OV25, NCT02477644), the olaparib and BV combination was studied in a maintenance setting after first-line chemotherapy in HGSOC patients. In the overall cohort, the combination of BV and olaparib resulted in a median PFS of 36.5 months compared to the 32.6 months in the placebo and BV combination [HR 0.78, CI (0.64–0.95), *p* = 0.0125]. The biggest benefit gained was in the *BRCA-*mutant cohort where the median PFS has not been reached versus 45 months for placebo and BV (HR 0.53, CI 0.34–0.83). The next best group to benefit was the HRD cohort at 50.3 versus 35.3 months for placebo (HR 0.56, CI 0.41–0.77) comparatively the HRD negative cohort performed the worst at 24.4 vs. 26.4 months (HR 1.04, CI 0.77–1.42) (Martín et al., 2020). The combination of PARPi and BV offers a benefit in extending PFS, especially in *BRCA*-mutant and HRD HGSOC, however the magnitude of clinical benefit in HR proficient cohorts is less clear. The phase II MITO25 study (NCT03462212) investigating rucaparib and BV in a maintenance setting in HRD and HR proficient newly diagnosed HGSOC and endometrioid patients is currently recruiting and results may confirm the benefit of this combination in the HR proficient setting.

The pan-VEGFR and PDGFR tyrosine kinase inhibitor Cediranib is another antiangiogenic agent being investigated with PARPi. In a phase II trial with olaparib and cediranib in relapsed platinum-sensitive HGSOC or endometrioid ovarian cancer, a PFS advantage was observed in the combination arm with a median PFS of 16.5 months compared to the olaparib only arm with 8.2 months (HR 0.50, CI 0.30–0.83, *p* = 0.006) (Liu et al., 2014; Liu et al., 2019). The overall cohort did not show a significant OS difference between treatment arms (44.2 versus 33.3 months, HR 0.64, CI 0.36–1.11, *p* = 0.11), and similarly the *BRCA*-mutant cohort did not have a significant difference in PFS (16.4 versus 16.5 months, HR 0.76, CI 0.38–1.49, *p* = 0.42) or OS (44.2 versus 40.1 months, HR 0.86, CI 0.41–1.82, *p* = 0.70) between combination and olaparib only arms. Comparatively, women in the *BRCA*-wildtype cohort had a significant improvement in the combination arm versus the olaparib only with PFS at 23.7 versus 5.7 months (HR.0.31, CI 0.15–0.66, *p* = 0.0013). The OS for this cohort was also significantly improved at 37.8 versus 23.0 months (HR 0.44, CI 0.19–1.01, *p* = 0.047) (Liu et al., 2014; Liu et al., 2019). This phase II trial suggested that women with *BRCA*-wildtype HGSOC derived the most benefit from this regimen.

However, this combination was also studied in the randomised NRG-GY004 phase III trial (NCT02446600) comparing patients with platinum sensitive recurrent high-grade serous or high-grade endometrioid ovarian, primary peritoneal, or fallopian tube cancers. Patients were screened for HR and LOH status with the BROCA-HR targeted next generation sequencing assay on germline and tumour DNA in 491 of 565 patients and compared across treatment arms of olaparib only, chemotherapy only and combination cediranib and olaparib. The HR-deficient cohort did the best compared to the HR proficient cohort, with a median PFS of 13.7 vs. 8.3 months (HR 0.41, *p* < 0.0001). When compared across treatment arms the cediranib and olaparib combination extended PFS to 20.4 months (HR 0.55, 95% CI 0.32–0.95) compared to 12.3 months in the chemotherapy arm and 13.1 months in olaparib only arm (HR 0.78, 95% CI 0.48–1.27). There was no difference between treatments in the HR proficient cohort, with a median PFS of 8.5 months in the cediranib and olaparib combination arm (HR 0.93 m, CI 0.68–1.27), and 9.0 months in the chemotherapy arm and 6.4 months in the olaparib only arm (HR 1.56, CI 1.15–2.12). This study also looked at LOH as a prognostic factor and found it was not predictive of response to olaparib, combination cediranib/olaparib or chemotherapy possibly suggesting that the HRD assay used was not sufficiently discriminatory (Swisher et al., 2021).

The phase II single arm trial EVOLVE interrogated PARPi-resistant HGSOC patients with a combination of cediranib and olaparib to identify objective response rates in PARPi-sensitive (PS), PARPi resistant (PR) and in patients who had chemotherapy post-PARPi progression (PE). A total of 34 patients were enrolled, with 9/11 PS, 8/10 PR and 7/13 PE patients with *BRCA1/2* mutations. Additionally, out of the 34 patients, four had a partial response to treatment and 18 patients were noted with stable disease. Of the cohorts 54.5% of PS patients (31.8–93.6), 50% of PR patients (26.9–92.9) and 36% of PE patients (15.6–82.8) reached the 16-weeks PFS with OS at 1 year 81.8% (61.9–100) in PS, 64.8% (39.3–100) in PR and 39.1% (14.7–100) in PE (Lheureux et al., 2019b). This study establishes that using patient response to PARPi could determine patient response to cediranib and PARPi combinations and suggests that this combination could have potential in PARPi-resistant disease.

Overall, the results from these clinical trials establish that there may be an OS benefit to be gained from the combination of cediranib and PARPi in the treatment of HGSOC, however results are inconclusive and are being investigated further in the phase III ICON9 trial assessing cediranib and olaparib vs. olaparib alone as a maintenance therapy in platinum sensitive recurrent OC currently enrolling and the phase II/III GY005 platinum-resistant relapsed OC cediranib PARPi combination therapy clinical trial (NCT02502266) (Lee, 2018; Elyashiv et al., 2021).

The triple combination of PARPi, bevacizumab and anti-PD-L1 therapy has also been trialed in the clinic. The phase II open-label study (NCT03574779) of dostarlimab, BV and niraparib in platinum-resistant recurrent EOC resulted in a median PFS of 7.6 months, disease control rate (DCR) of 76.9% with 23 patients with stable disease, seven patients with PRs and no CRs (Liu et al., 2021). The clinical side effects were tolerable however 34.1% of patients discontinued one of the three drugs due to adverse events. Other trials ongoing include the phase II DUO-O study investigating durvalumab, olaparib and BV after treatment with carboplatin, paclitaxel and BV (AGO-OVAR23/ENGOT-OV46, NCT3737643) and the phase II study combining nivolumab, rucaparib and BV in recurrent ovarian cancer. The MEDIOLA study also compared treatment of olaparib and durvalumab (O + D) to O + D and BV (O + D + BV). The O + D + BV cohort had better a ORR at 77% (95% CI 58.9%–90.4%) compared to 31.3% (95% CI 16.1%–50.0%) in the O + D cohort. This was reflected in the PFS with the triple combination eliciting a PFS of 14.7 months compared to 5.5 months. Both treatments were tolerable however the triplet combination had a higher rate of patients discontinuing treatment, 17% versus 6% in the O + D cohort (Drew et al., 2020). The triplet therapy of PARPi, bevacizumab and CI seems to elicit more durable responses compared to PARPi and CI alone, however the tolerability of this treatment long term is unclear.

#### 5.2.3 PARPi and chemotherapy

In recent years, studies have demonstrated platinum compounds can act as immune modulators effectively inducing immunogenic cell death alongside their DNA-damaging characteristics (de Biasi et al., 2014; Rébé et al., 2020). Platinum chemotherapies were primarily known as DNA-damaging agents that HR-deficient tumours readily respond to as they interfere with DNA transcription and replication. This leads to DNA damage and subsequent activation of DNA repair pathways which in HR-deficient cells, induces cell death (Martin et al., 2008). However, there is variation between platinum agents in their ability to augment immune responses, some can promote anti-tumour immune responses through the recruitment of effector cells, upregulation of MHC molecules and downregulation of immunosuppressive factors (de Biasi et al., 2014). The use of PARPi in combination with platinum chemotherapy has the potential to sensitise tumour cells to DNA-damaging agents and potentially the anti-tumour immune response (Nguewa et al., 2006; Cheng et al., 2013). However, PARPi effects on DNA repair enhances chemotherapy-induced myelosuppression, creating a major concern in patient tolerability to this combination therapy (Dent et al., 2013). The overlapping toxicities affect dosing and scheduling, resulting in attenuated doses of either or both PARPi and platinum therapeutics, potentially affecting the efficacy of either drug, due to the use of concentrations below the recommended monotherapy dose. The recent development of PARP-1-specific PARPi may provide new opportunities (Johannes et al., 2021).

A phase I study (NCT00516724) trialled olaparib with paclitaxel or carboplatin or carboplatin or the paclitaxel (CP) combination in advanced solid tumours refractory to standard treatments (van der Noll et al., 2020a). Patients treated with daily olaparib continuously in combination with CP experienced hematologic toxicities resulting in the attenuated scheduling (van der Noll et al., 2020a). Patients receiving intermittent olaparib increased tolerability but still experienced significant myelosuppression (van der Noll et al., 2020b). However, results from this trial did identify two olaparib treatment schedules that were tolerable in patients. Further interrogation of the olaparib and CP combination in study 41(NCT01081951) in platinum sensitive recurrent ovarian cancer achieved a significant improvement in PFS in the combination arm compared to chemotherapy alone (12.2 versus 9.6 months; HR 0.51, CI 0.34–0.77, *p* = 0.0012). The combination was well tolerated with only 15% reporting adverse events in the combinational group versus the 21% in the chemotherapy group alone. Most benefit was assumed derived from the maintenance phase of olaparib and specifically in the *BRCA1/2* mutant cohort. Regardless, the ORR was 64% versus 58% between the different treatment arms.

Veliparib is a relatively weaker PARP trapper therefore potentially better tolerated for combination studies. Veliparib has been trialed with the standard carboplatin and paclitaxel (CP) chemotherapy combination in the phase III VELIA study in women with newly diagnosed HGSOC (Coleman et al., 2019; Aghajanian et al., 2021). Patients were to receive 6 cycles of CP following primary cytoreduction or with an interval cytoreduction. Veliparib or placebo was administered during CP at an attenuated dose of 150 mg twice daily and subsequently a full dose at 400 mg twice daily after CP treatment. Of the 1,140 patients enrolled, 26% were *BRCA-*mutant and 55% noted as HRD. Overall, the addition of Veliparib significantly improved PFS with median PFS of 23.5 months vs. 17.3 (HR 0.68%, 95% CI [0.56,0.83], *p* < 0.001] for Veliparib vs. placebo. The greatest PFS benefit was seen in *BRCA-*mutant and HRD cohorts at 34.7 months vs. 22.0 months (HR 0.44, 95% CI [0.28,0.68], *p* > 0.001) and 31.9 months vs. 20.5 months (HR 0.57, 95% CI [0.43,0.76], *p* > 0.001) respectively. Veliparib has currently not been approved for use in the treatment of OC.

Another chemotherapy agent that has been generating interest is low-dose cyclophosphamide (LDCy), it is a potent immunostimulant when delivered at low doses and is well tolerated, eliciting clinically beneficial responses in roughly 44% of recurrent OC cases (Handolias et al., 2016). Studies have shown that LDCy promotes anti-tumour immunity through the selective depletion of Tregs and enhancing the function of effector T cells (Handolias et al., 2016; Madondo et al., 2016). A phase II clinical trial (NCT01306032) explored the combination of veliparib and cyclophosphamide in recurrent *BRCA*-mutant OC and HGSOC (Kummar et al., 2015). The addition of the low dose or 60 mg of veliparib to 50 mg of LDCy had no improvement in ORR or median PFS compared to LDCy monotherapy. Stratifying patients according to BRCA status and DNA repair defects also did not predict response to either monotherapy or combination. But, two patients from this trial had a prolonged clinical benefit from the combination treatment, receiving over two years of treatment, which was still ongoing at the time of data analysis (Kummar et al., 2015). This trial encompassed not only HGSOC but primary peritoneal, fallopian tube or *BRCA-*mutant OC which could have affected the ability to decipher characteristics that determine patient responses. Additionally, the doses of veliparib used were below the standard 250–400 mg, thus higher doses of veliparib may yield more significant results or alternatively the addition of a third drug could potentially boost responses. Although their correlative studies could not identify characteristics that determine patient prognosis to treatment further studies could possibly interrogate features of patients that respond to this combination to broaden treatment cohorts.

The NCT02853318 phase II clinical trial observed the effects of pembrolizumab (anti-PD1), bevacizumab and LDCy in recurrent OC (Zsiros et al., 2021). The triple combination had an ORR of 47.5% and a median PFS of 10 months, with 100% of platinum-sensitive patients meeting the 6-month PFS rate compared to only 59% of the platinum-resistant patients (*p* = 0.024). Combining LDCy with PARPi and CI could elicit similar responses, targeting cancer cells and invigorating immune responses, particularly in platinum-sensitive/HRD patients that derive the most benefit from these drugs.

The combination of olaparib and LDCy has been examined in recurrent OC and triple negative breast cancer to determine its safety and tolerability (Lee et al. Br J Cancer). A tolerable regimen was identified and in HGSOC and the *gBRCAm* subset, the unconfirmed objective RR was 48% and 64% respectively.

## 6 Conclusion

The treatment of HGSOC provides an ongoing challenge, due to the heterogenous and metastatic nature of this disease rendering women susceptible to disease relapse. Extensive research performed to characterise the disease has led to a better understanding of which characteristics correlate with clinical benefit from current therapeutic regimens. However, monotherapies do not effectively target the multiple aspects of HGSOC tumours that can occur simultaneously, in the vast majority of cases. The development of drug resistance is a growing concern, especially following treatment with PARPi and thus the use of combination regimens has garnered increasing interest. The characterisation of PARPi has elucidated its myriad roles in DNA repair and regulation, including roles in chromatin remodelling and methylation. Additionally, effects of PARPi on immune cells and immune responses offer alternative pathways for therapeutic exploitation. To date we have seen PARPi combined with chemotherapy, angiogenesis agents, immune checkpoint inhibitors and more recently novel therapeutics including epigenetic drugs and other DNA repair inhibitors, with some trials investigating triple combination treatments. The benefit of these therapies for women with platinum-resistant and HR proficient OC are still unclear when compared to their platinum-sensitive and HRD OC counterparts. One of the most challenging aspects of combination therapy is tolerability. Investigating alternative treatment sequencing and scheduling could result in triple-combination therapies becoming more tolerable and in prolongation of survival if successfully matched to the molecular characteristics of the HGSOC. Additionally, clinical trials involving correlative studies to investigate outcomes are essential to establish ideal biospecimen cohorts to enable the most complete understanding of a trial outcome and for further research. Understanding the characteristics that drive responses could improve strategies for driving prolonged remissions and ultimately improve the survival outcomes for women with HGSOC.
